# Development and validation of hybrid machine learning approach for predicting survival in patients with cervical cancer: a SEER-based population study

**DOI:** 10.3389/fonc.2025.1605378

**Published:** 2025-06-18

**Authors:** Anjana Eledath Kolasseri, Venkataramana B.

**Affiliations:** School of Advanced Sciences, Vellore Institute of Technology, Vellore Tamil Nadu, India

**Keywords:** cervical cancer, SEER database, machine learning, survival models, hybrid models

## Abstract

**Background:**

Accurate survival prediction in cervical cancer is crucial for personalized therapy, particularly in high-risk groups where early intervention might enhance results. The study aims to create a hybrid survival model that integrates Cox Proportional Hazards (CoxPH) with Elastic Net regularization and Random Survival Forest (RSF) to improve prediction accuracy and interpretability.

**Methods:**

Data from the SEER database (2013–2015) were pre-processed through normalization and encoding. RSF recorded non-linear interactions between covariates, while the CoxPH Elastic Net Regularization model provided linear interpretability and identified key variables. Model parameters were optimized using cross-validation, and final performance was assessed on an independent test set using metrics including C-index, Integrated Brier Score (IBS), AUC-ROC, and calibration plots.

**Results:**

The hybrid model outperformed the individual models with an Integrated Brier Score (IBS) of 0.13 and a concordance index (C-index) of 0.82. With an AUC-ROC of 0.84, the model provided robust calibration and classification performance on the independent test set, effectively separating between individuals at high and low risk.

**Conclusion:**

The hybrid model provides a promising tool for personalized risk stratification in cervical cancer based on survival probability. Further testing in varied clinical categories is recommended to confirm its efficiency in precision oncology.

## 1 Introduction

Cervical cancer (CC) is one of the most common types of cancer that affect women all over the world, especially in developing countries, and is still a major cause of cancer-related deaths (1). Despite developments in screening and immunization programs, it remains a significant health burden, particularly in areas with restricted access to healthcare facilities (2). The prognosis of cervical cancer is highly dependent on timely diagnosis and treatment, with tumor stage, lymph node involvement, metastasis, and socioeconomic status all having major roles in patient survival (3). As a result, the analysis of survival is critical in interpreting the impact of these variables and predicting outcomes in cervical cancer patients. Accurate survival projections can inform treatment decisions and enable doctors to personalize therapy to improve patient outcomes (4).

To address this, various kinds of statistical models for analyzing survival data have been designed, including the Cox Proportional Hazards (Cox PH) model, which is popular due to its interpretability and theoretical approach. The Cox model is a semi-parametric model that follows the proportional hazards assumption, making it adaptable to situations in which the hazard ratio between groups remains constant throughout the time interval (5). However, this assumption may not always be valid in complicated illnesses such as cervical cancer, where the link between variables and survival may differ drastically between phases of the disease (6). More flexible modeling techniques are needed to account for the intricate relationships between tumor stage, lymph node involvement, treatment methods, and patient demographics like age and socioeconomic status. This is where machine learning algorithms, specifically Random Survival Forests (RSF), have shown their effectiveness. RSF is a non-parametric approach that can capture complex, non-linear relationships without assuming anything about the hazard function (7). Nevertheless, RSF models often lack transparency, leading to challenges in interpreting their predictions in a clinical environment (8).

A hybrid method that combines traditional models with machine learning techniques is important due to the complex nature of cervical cancer survival data (9). In this research, we suggest a mixed model that combines the Cox and RSF models to enhance survival forecasts’ precision and explanatory power. The combination method utilizes RSF’s adaptability to capture non-linear relationships while preserving the interpretability of Cox models, giving important hazard ratios and parametric insights.

Our goal is to overcome the limitations of each method by combining these models. This hybrid method is utilized on an extensive dataset of cervical cancer patients to offer improved and detailed survival forecasts while maintaining results that are understandable for clinical decision-making.

This article discusses creating, executing, and confirming a hybrid model to enhance survival predictions and determine key predictors of patient outcomes in cervical cancer.

## 2 Materials and methods

### 2.1 Data sources and inclusion criteria

The study analyzed cervical cancer data from the Surveillance, Epidemiology, and End Results (SEER) database, National Cancer Institute (https://seer.cancer.gov/), a free US cancer registry from 2013 to 2015. We obtained access to the SEER database files, and all writers conformed to SEER database policies during the search procedure. Individual informed consent was not essential because personal information was not utilized in this investigation. According to SEER data use standards, IRB permission was not required for this study since it used de-identified, publicly available data from the SEER program.

The inclusion period was chosen to provide an adequate follow-up time for survival analysis, with a minimum of five years from diagnosis to assess outcomes. The study consisted of patients who had been diagnosed with primary cervical cancer using the International Classification of Diseases for Oncology, Third Edition (ICD-O-3) codes. To maintain the integrity of the analysis, only patients who provided complete data on essential clinical and therapy characteristics were included.

The variables in this study include a wide variety of clinical, therapeutic, demographic, and socioeconomic aspects, which are critical for understanding cervical cancer survival outcomes. The clinical factors include T stage (tumor size and extent), N stage (lymph node involvement), M stage (presence or absence of distant metastases), and Overall Stage (Localized, Regional, or Distant). Treatment-related variables include whether the patient received radiation therapy (binary: 1 = received, 0 = not received) and chemotherapy (binary: 1 = received, 0 = not received), as well as months from diagnosis to treatment, which measures the time between diagnosis and treatment start. Also, Patients who lived longer than 60 months from diagnosis were considered alive (10).

Demographic characteristics include age (at the time of diagnosis), race (White, Black, and Other, which includes American Indian/Alaskan Native and Asian/Pacific Islander), and marital status (Married, Single, Divorced, or Widowed). Household Income is used to determine socioeconomic level, with categories including <$50,000, $50,000-$74,999, and $75,000+. The outcome variables are Survival Months, which represent the number of months the patient lived after diagnosis, and Vital Status, a binary variable that shows whether the patient died (1) or was alive (0) after the research period.

Cases with missing or limited data for clinical, therapeutic, or survival factors were excluded. This was done to ensure accurate and relevant survival analysis, as missing data might result in bias. The chosen date (2013-2015) corresponds to a period when modern treatment options, such as advanced radiation techniques and combination chemoradiotherapy, were more standardized, giving a meaningful backdrop for assessing current survival results in cervical cancer patients.

### 2.2 Data preprocessing

Data pretreatment stages included managing missing values, normalizing continuous variables, and encoding categorical characteristics. Multiple imputations were used to decrease bias while retaining statistical power in missing data, particularly in demographic characteristics like age and tumor size. Basic techniques that relied on the type of attribute were used to impute missing values: the mode for categorical variables and the mean for continuous variables. Furthermore, rows with excessive missingness across multiple features were excluded from the analysis. Simple imputation was considered adequate because of the low percentage of missing data and the model’s strong validation on independent test data, even though there are more advanced imputation techniques available. Continuous features, such as tumor size and patient age, were standardized to have a mean of zero and a standard deviation of one, which is crucial for the proper implementation of regularization methods such as Elastic Net (11). Categorical variables, such as treatment type, were one-hot encoded before being included in the model. The dataset was randomly divided into training (70%) and independent test sets (30%) to guarantee that model performance could be assessed on unknown data.

## 3 Model development

### 3.1 Cox proportional hazards model with elastic net regularization

The Cox Proportional Hazards (Cox PH) model is commonly used in survival analysis to obtain the connection between variables (such as patient age, tumor size, and therapy type) and time to an event (e.g., death or disease progression) (12). Elastic Net regularization was used in the Cox PH model to handle high-dimensional data with many covariates and accomplish feature selection by shrinking coefficients for less significant variables (13). Elastic Net regularization enhances interpretability by focusing on the most relevant variables, making the model especially beneficial when feature selection is important. It improves stability by reducing overfitting, particularly when the dataset contains multiple confounders.

#### 3.1.1 Elastic net regularization

It combines L1 (Lasso) and L2 (Ridge) penalties. L1 regularization aids in feature selection by reducing some coefficients to zero, removing unimportant characteristics. L2 regularization reduces coefficients without eliminating any variables, reducing multicollinearity and increasing model robustness to overfitting (14). The mixing parameter (alpha), which defines the balance between L1 and L2 penalties, and the regularization strength (lambda), were improved using 5-fold cross-validation on the training set. This ensures that the model doesn’t overfit or underfit the data (15, 16).

Risk Score Generation: The Cox PH Elastic Net Regularization model gives a risk score for each patient, which represents their relative hazard. These risk ratings enable the algorithm to rank patients based on their likelihood of having the event, with higher scores indicating more risk.

### 3.2 Random survival forest

Random Survival Forest (RSF) is a nonparametric ensemble approach that combines the conventional random forest algorithm with survival analysis (7). RSF is very effective at dealing with complicated, non-linear correlations between variables and survival outcomes, making it a useful addition to Cox PH’s linearity (17). RSF is adaptable to high-dimensional data and can simulate covariate interactions without explicit specification (18). RSF calculates the time-dependent survival probability for each patient. These probabilities describe the possibility of living beyond a specific time point, and they can account for complex relationships between factors that Cox PH may not capture. Also, it determines variable significance measures to determine the relative relevance of each covariate in predicting survival. This is especially beneficial for discovering important prognostic features in complex datasets.

RSF improves the hybrid model by enabling greater flexibility in modeling non-linear interactions and managing high-dimensional data with large covariate interactions (18). It helps Cox PH by detecting patterns that a linear model may overlook, such as complex relationships between treatment modalities and patient characteristics (19).

### 3.3 Hybrid model (combining Cox PH with elastic net regularization and random survival forest)

To develop the hybrid model, the predictions from Cox PH with Elastic Net Regularization and Random Survival Forest were combined to get the strengths of both approaches. To improve prediction accuracy, all models were combined using a custom ensemble approach. This was performed using a linear regression weighting procedure that compared each model’s prediction to its actual predicted value. The weighting coefficients were then used to weight the ensemble prediction, which is an average result. The custom ensemble was predicted to obtain better results than any other approach, and it was easy to implement and adapt to model changes (20).

A weighted average of the risk scores from the two models was obtained. The final weights for the hybrid model (70% CoxPH Elastic Net Regularization, 30% RSF) were identified through grid search optimization. Multiple weight combinations (in 10% increments) were evaluated using 5-fold cross-validation, and the combination yielding the highest average concordance index (C-index) on the validation folds was selected. It ensured performance-based weight allocation. The last hybrid prediction was:


$$\begin{array}{c} \text{Hybrid Prediction }\\ =\;w_{1}\times \text{ Cox PH Risk Score }+\;w_{2}\;\times \text{ RSF Survival Probability} \end{array}$$

$w_{1},w_{2}$ are the weights applied to each model. These weights were calculated using 5-fold cross-validation to balance the contributions of both models and reduce prediction error.

While Cox PH Elastic Net Regularization detects linear relationships and selects significant variables, RSF captures nonlinear interactions and is more adaptable to modeling complicated data. By integrating both models, the hybrid model achieves the interpretability and robustness of Cox PH while maintaining the flexibility and prediction accuracy of RSF.

### 3.4 Model evaluation and validation

#### 3.4.1 Cross validation

On the training set, the individual models (Cox PH Elastic Net Regularization model and RSF) as well as the hybrid model were tested using 5-fold cross-validation. During cross-validation, the C-index was produced to measure each model’s ability to rank patients based on survival risk (21). The Integrated Brier Score (IBS) was calculated as well to obtain the overall predictive accuracy of survival probability (22).

#### 3.4.2 Performance metrics

Concordance Index: The C-index evaluates the models’ discriminative abilities by comparing predicted risk scores to survival times. Higher values suggest improved discriminatory performance. It varies from 0.5 (no better than random chance) to 1.0 (perfect prediction). In clinical research, a C-index greater than 0.7 is considered to be good, but values greater than 0.8 indicate high discriminative performance (23).

Integrated Brier score: The Integrated Brier Score (IBS) assesses the overall accuracy of predicted survival probabilities over time, accounting for both discrimination and calibration. It ranges between 0 (perfect prediction) and 0.25 for binary outcomes with a 50% occurrence rate. Lower IBS values suggest improved model performance, and values less than 0.2 are generally considered acceptable in clinical survival models (22).

Calibration Plots: Calibration plots were created to compare predicted survival probability with observed survival rates over several periods. Well-calibrated models will produce predictions that are closely aligned with the 45-degree line (24).

Mean Absolute Error (MAE): MAE calculates the average absolute error between predicted and observed survival periods. It measures the total variance in survival time forecasts, with lower MAE values showing higher prediction accuracy (25).

Mean Squared Error (MSE): MSE measures the squared difference between predicted and actual survival times. MSE is a more sensitive measure to larger errors than MAE since it penalizes larger deviations more strongly. Lower MSE values indicate higher prediction accuracy, particularly when avoiding significant prediction errors (25).

Survival Accuracy: Survival accuracy is the proportion of patients accurately identified as “event” or “censored” at time intervals. It examines the model’s classification performance in survival cases, evaluating accuracy at time points such as 1, 3, and 5 years after diagnosis. Higher survival accuracy means that the model performed better at properly categorizing survival status over time (26).

#### 3.4.3 Independent test set evaluation

Following cross-validation, the resulting hybrid model was evaluated on an independent test set (30% of the data) that was excluded from model development. The independent test set gave an unbiased assessment of model generalization. The test set was evaluated on C-Index, IBS, MAE, MSE, survival accuracy, and calibration plots.

#### 3.4.4 Statistical software and implementation

All analyses were conducted using R (version 4.0) and Python (version 3.8).

## 4 Results

The final cohort included 3810 cervical cancer patients, with a median age of 27.93 years and a median survival period of 59.05 months. **Table 1** summarizes all 14 of the important attributes that were selected, including the objective “Survival months,” and consists of both continuous (numeric) and categorical (discrete) variable types. The primary event (death) affected 30% of the cohort, with the remaining patients being censored at the conclusion of the research.

**Table 1:** Description of selected clinical, demographic, and treatment variables used for analysis.

No.	Attribute	Description	Type
1	Age	Age at time of diagnosis.	Numeric
2	Stage	Stage of tumor – based on T, N, and M.	Categorical
3	T stage	AJCC component describing tumor size.	Categorical
4	N stage	AJCC component describing lymph node involvement.	Categorical
5	M stage	AJCC component describing tumor dissemination to other organs	Categorical
6	Radiation Therapy	Indication of whether the patient has received radiation therapy	Categorical
7	Chemotherapy	Indication of whether the patient has received chemotherapy	Categorical
8	Race	Race of the individual	Categorical
9	Marital status	Indication of the marital status of the individual	Categorical
10	Household income	Indication of household income of the individual	Categorical
11	Tumor size	Measurement of tumor size.	Numeric
12	Months from diagnosis to treatment	time interval between a patient’s initial diagnosis and the beginning of their treatment.	Numeric
13	Vital status	Indication of whether the patient is alive or dead	Categorical
14	Survival months	Number of months that patient is alive from date of diagnosis.	Numeric

### 4.1 Model performance on training and validation sets

The data was divided into 80% for training and 20% for testing. Predictive models were fitted using cross-validation and evaluated on the train data for accuracy, recall, F1-measure, sensitivity, and specificity.

The Cox PH model, with Elastic Net regularization, was tested for its ability to predict survival outcomes. Cross-validation was used to find the optimal penalty parameter, lambda (0.11), that reduced prediction error while balancing feature selection and model complexity. The chosen lambda allowed the model to keep significant characteristics while regularizing others, lowering the risk of overfitting. The Cox model was assessed on the test dataset using Mean Absolute Error (MAE), Mean Squared Error (MSE), and Survival Accuracy. The MAE, which measures the average deviation between predicted survival risk scores and observed outcomes, was rather low, indicating that the model provided reliable predictions. The MSE was somewhat greater, indicating exposure to outliers or significant errors. Finally, survival accuracy, as tested at a probability threshold, demonstrated that the Cox model correctly identified survival outcomes in many test cases.

These findings suggest that the Cox Elastic Net Regularization model is most successful when the connection between predictors and survival outcomes is especially linear. However, the model’s ability to capture complicated, non-linear patterns found in high-dimensional clinical data may be limited, highlighting the importance of complementing modeling techniques. **Table 2** displays the non-zero coefficients for the important prognostic variables in the Cox PH with Elastic Net Regularization model. It chose a minimal group of predictors from the entire variable set, keeping only those that contributed significantly to the prediction of survival risk. Variables with coefficients reduced to zero were omitted from the final model.

**Table 2:** Variables having non-zero coefficients from the Cox PH model with elastic net regularization.

Variables	Cox PH with Elastic Net Regularization Co-efficients
TstageT4	0.354
MstageM1	0.253
Household income (<$44000)	0.133
NstageN1	0.116
Tumor size	0.0011
Stage (Localized)	-0.244
Marital status (Married)	-0.064
Household income (>$75000)	-0.082
Overall Risk score	0.762

Variables with coefficients reduced to zero were omitted from the final model.

Among the retained features, T stage 4 exhibited the highest positive coefficient (0.354), showing a substantial link to increased risk. Similarly, M stage 1 (coefficient = 0.253) and N stage 1 (0.116) were also related to an increased probability of death, which is consistent with clinical predictions for metastatic dissemination. A negative coefficient (-0.244) was found for Stage: Localized, showing a protective impact as compared to more advanced stages. Married (coefficient = -0.064) and having a family income of more than $75,000 (-0.082) were shown to be linked with decreased risk, whereas household income less than $44000(0.133), which shows the importance of socio-demographic variables in cervical cancer survival. The variable “risk score”, generated internally by the model, had the highest coefficient (0.762), reflecting the combined linear contribution of all retained features to the overall survival risk estimate.

The RSF model was developed to capture non-linear interactions and interactions between variables that the Cox model may not effectively handle. To improve the RSF model, we performed hyperparameter tuning on critical parameters such as the number of trees, the number of variables chosen at each split, the minimum node size, and the splitting procedure. The log-rank splitting algorithm was utilized, which is specifically designed for survival analysis and allows the model to make divides based on survival times. After adjustment, the RSF model performed well on the test dataset. The MAE was comparable to that of the Cox model, indicating that the RSF model produced somewhat accurate predictions. Notably, the RSF model has a lower MSE than the Cox model, suggesting less significant deviations in its predictions. This shows that the RSF was better at handling cases with complicated interactions between variables because it was less influenced by outliers. The survival accuracy was slightly lower than that of the Cox model, implying that the Cox model classified survival outcomes more accurately.

The results of the RSF model demonstrate its ability to identify non-linear correlations between variables, which are common in clinical datasets. However, the model’s versatility may come at a cost of interpretability, as RSF does not give a clear framework for understanding how specific predictors affect survival. **Table 3** shows the 5 most significant prognostic factors for the RSF. The top predictors are listed based on their variable importance (VIMP) for the RSF. Higher VIMP values indicate greater importance of the variable in the model’s predictive power.

**Table 3:** VIMP values for RSF for five most important variables with Higher values indicating greater importance of the variable in the model’s predictive power.

Variable	Variable Importance (VIMP)
T-stage	0.047
Tumor Size	0.036
Stage	0.028
Household Income	0.025
Age	0.019

### 4.2 Hybrid model: combining Cox elastic net regularization and RSF survival predictions

The hybrid model was developed by combining predictions from the Cox Elastic Net regularization and RSF models using an ensembled weighted average technique, with weights set at 70% for the Cox model and 30% for the RSF. This weighting method was used to achieve an optimal balance between the Cox model’s interpretability and linearity and RSF’s nonlinear predictive ability (20). By selecting the Cox model, we maintained the focus on linear interactions while the RSF component captured additional data and complexity.

The hybrid model outperformed both separate models in the majority of the evaluation criteria. It had the lowest MAE, suggesting that the hybrid model produced the most reliable predictions with minimal average deviation from observed survival outcomes. The MSE was also significantly reduced indicating that the hybrid model was adaptable to major errors. This reduction in MSE indicates the model’s stability and implies that combining linear and nonlinear predictions improves exposure to outliers. In addition, the Cox model with elastic net regularization has slightly higher survival accuracy than the hybrid model since it directly predicts survival time and selectively highlights the most predictive variables, giving it an advantage in accurate survival predictions. In contrast, the hybrid model, while dominant across multiple metrics, has been optimized for overall robustness rather than precision in survival time, which may reduce survival accuracy. The evaluation metrics are shown in **Table 4**.

**Table 4:** Performance evaluation of all the survival Models, which indicates the hybrid model outperforms in numerous measures, except survival accuracy.

Model	C-Index	IBS	MAE	MSE	Survival Accuracy
Cox with Elastic Net Regularization	0.807	0.049	0.550	0.324	0.856
RSF	0.809	0.036	0.332	0.142	0.778
Hybrid Model	0.812	0.032	0.213	0.069	0.844

These findings indicate that the hybrid model effectively combines the complimentary advantages of Cox Elastic Net Regularization and RSF, resulting in better generalization and prediction accuracy. The hybrid method, which incorporates both linear and nonlinear interactions, provides an improved comprehension of survival outcomes, making it especially useful in complicated clinical contexts.

### 4.3 Survival curve and calibration plots

**Figure 1:**
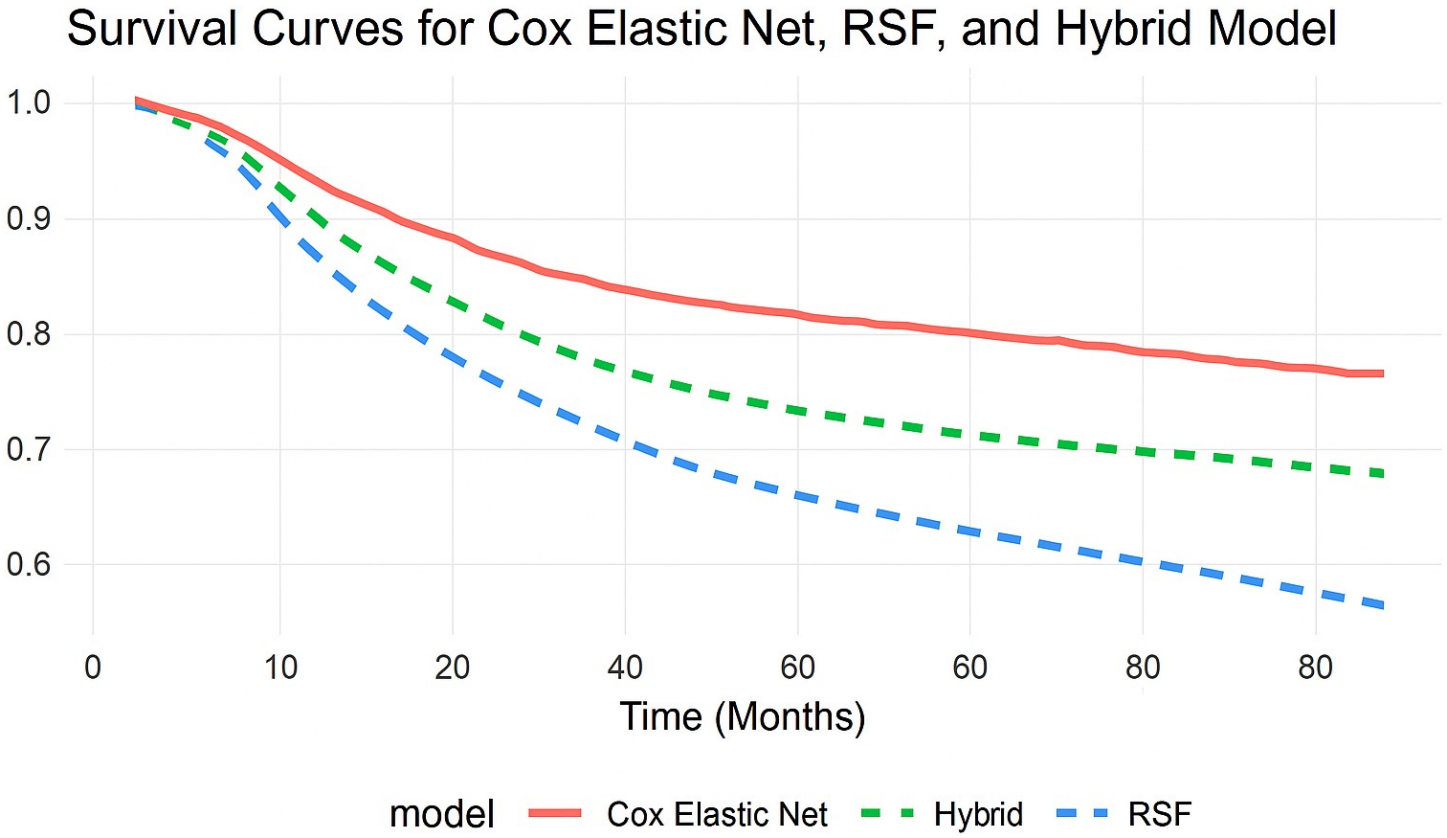
Comparison of predicted survival curves from Cox with Elastic Net Regularization, RSF, and Hybrid models. This comparison demonstrates the models’ ability to identify survival patterns while distinguishing long-term risk.

To visually assess the models’ prediction accuracy, survival curves were created for each model, illustrating predicted survival probabilities with time. The Cox Elastic Net model (solid red line) provides a more conservative survival estimate, whereas the RSF model (dashed blue line) forecasts greater reductions in survival probability. The Hybrid model (dashed green line) combines the two techniques and exhibits intermediate behavior, trying to achieve a balance between flexibility and interpretability. Also, the hybrid model’s survival curve closely matched the observed survival probabilities, implying that it generates the most accurate survival predictions throughout the full period (**Figure 1**).

**Figure 2:**
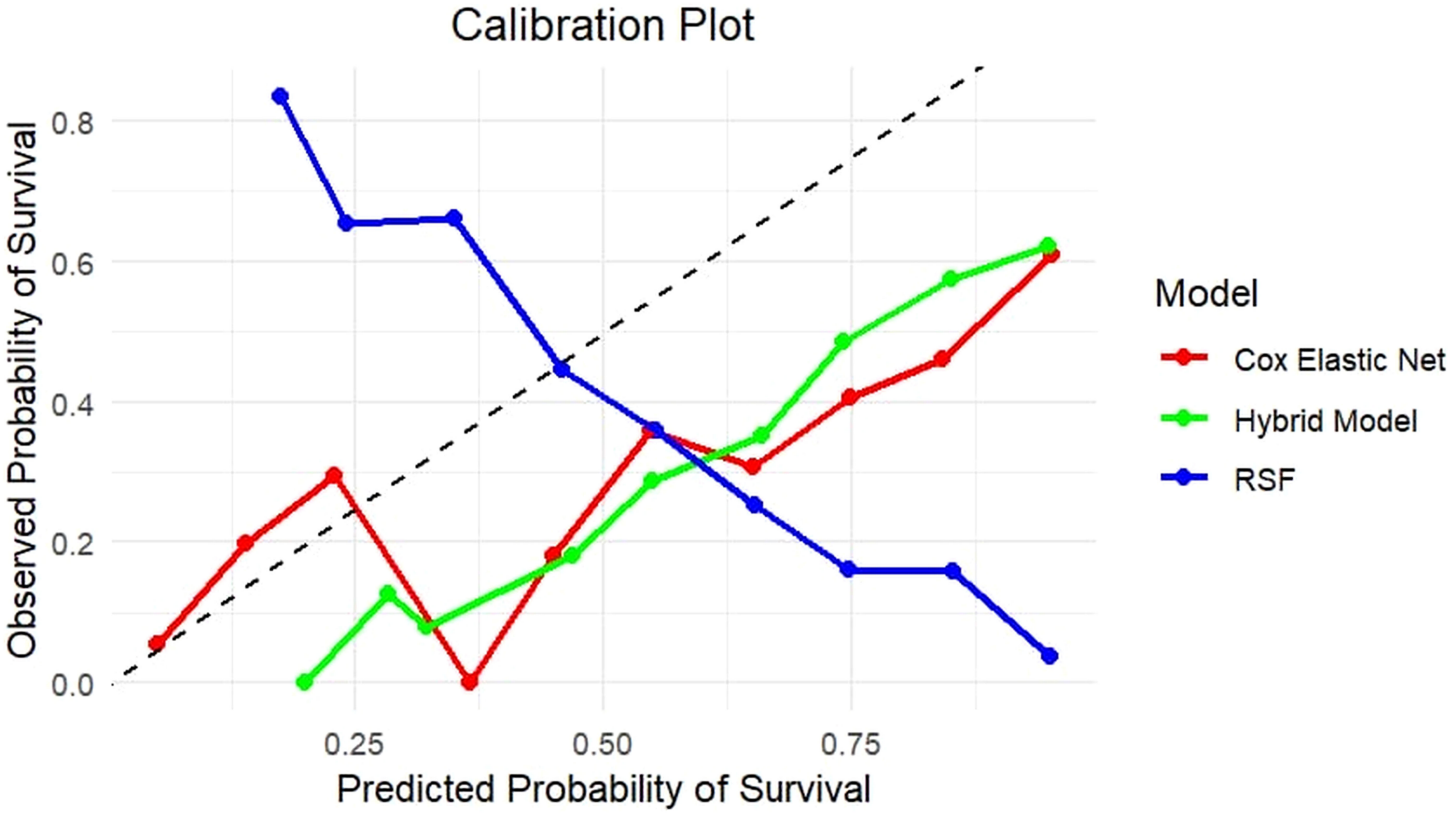
Calibration plot comparing predicted and observed survival probabilities for Cox with Elastic Net Regularization, RSF, and Hybrid models. The plot represents the association between predicted survival probability (x-axis) and actual survival outcomes (y-axis) across risk categories. The diagonal line indicates perfect calibration. The Hybrid model (green) closely follows the diagonal, showing better calibration throughout the probability range than the Cox Elastic Net Regularization (red) and RSF (blue), which show overestimation and underestimation in certain probability bins. This highlights the hybrid model’s ability to produce well-calibrated survival predictions.

Calibration plots were additionally used to determine how well predicted and observed survival probability was accepted. The calibration plot for the hybrid model indicated that its predictions were well-calibrated, closely matching observed probabilities, particularly at significant survival time periods (**Figure 2**). This indicates the hybrid model's benefit over the independent models since it provides accurate and well-calibrated predictions.

### 4.4 Independent test set evaluation

The hybrid model’s reliability was further tested on the independent test set, which contained 30% of the original dataset and was excluded from all training and validation steps. **Table 5** displays the metrics that showed the hybrid model’s higher generalization capabilities.

**Table 5:** Performance metrics on Independent test set for the hybrid survival model, which shows high performance than the individual models.

Metrics	Value
C-Index	0.82
IBS	0.029
MAE	0.197
MSE	0.064
Survival Accuracy	0.85

**Figure 3:**
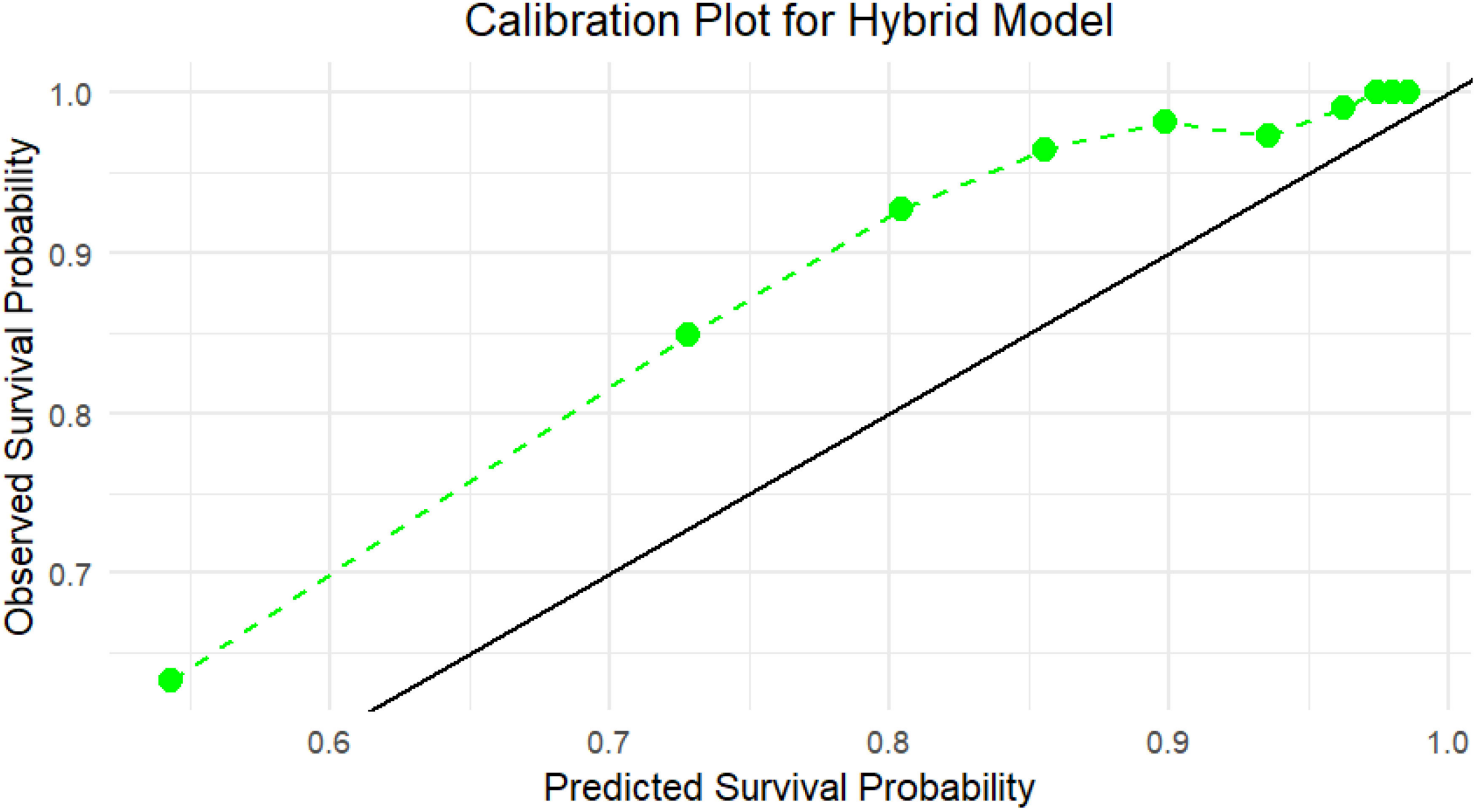
Calibration plot for the Hybrid model on the independent test set, which indicates the agreement between predicted survival probabilities (x-axis) and observed survival outcomes (y-axis) using test data. The green dashed line shows the Hybrid model’s calibration curve, while the black diagonal line denotes perfect calibration. The Hybrid model closely aligns with the ideal line, especially in the higher probability range (≥0.7), showing strong reliability and calibration of the model’s predictions in unseen data.

Calibration plots for the hybrid model on the test set indicated a good fit between predicted survival probabilities and observed outcomes, with calibration curves close to the 45-degree line (**Figure 3**). This alignment proved that the hybrid model made well-calibrated survival predictions, demonstrating its reliability in real-world clinical settings.

**Figure 4:**
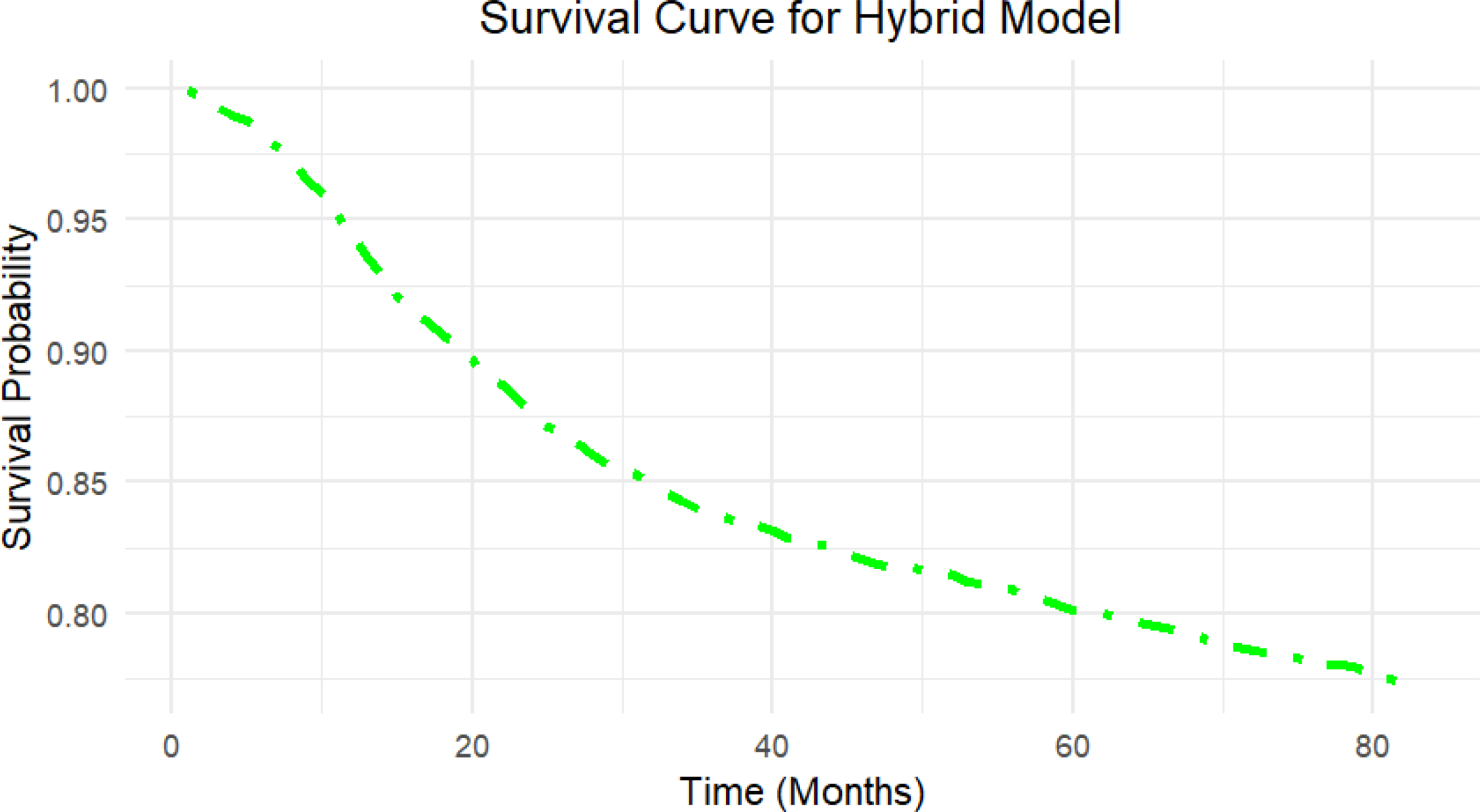
Survival curve generated by the Hybrid model on the independent test set. The curve illustrates the predicted survival probability over time (in months) for the independent test set using the Hybrid model, which highlights the model’s capacity to provide clinically relevant survival outcomes for patient risk stratification.

The hybrid model’s survival curve shows that it performs well in predicting a reasonably steady decline in survival probability over time, rather than a sudden drop-off (**Figure 4**). The curve’s smooth and continuous decrease indicates that the hybrid model properly represents a modest and consistent risk rise over time, which corresponds to the nature of survival data in this situation.

### 4.5 Feature importance in hybrid model

To further understand the factors influencing survival predictions, we used a feature importance analysis that included information from the Cox Elastic Net Regularization and RSF models. In the Cox model, feature relevance was determined by the size of the coefficients, with larger values suggesting stronger linear relationships with survival. The Cox model identified the most relevant characteristics as T stage, household income, M stage, etc., which were important because of their strong linear connection with survival outcomes.

In contrast, the RSF model’s feature importance was determined using permutation importance, which evaluates the increase in prediction error that results from randomly permuting the values of a particular feature. This approach is useful for identifying factors having nonlinear or interaction effects on survival. The RSF model identified T stage, Stage, N stage, etc. as important predictors, highlighting the model’s sensitivity to nonlinear relationships.

**Figure 5:**
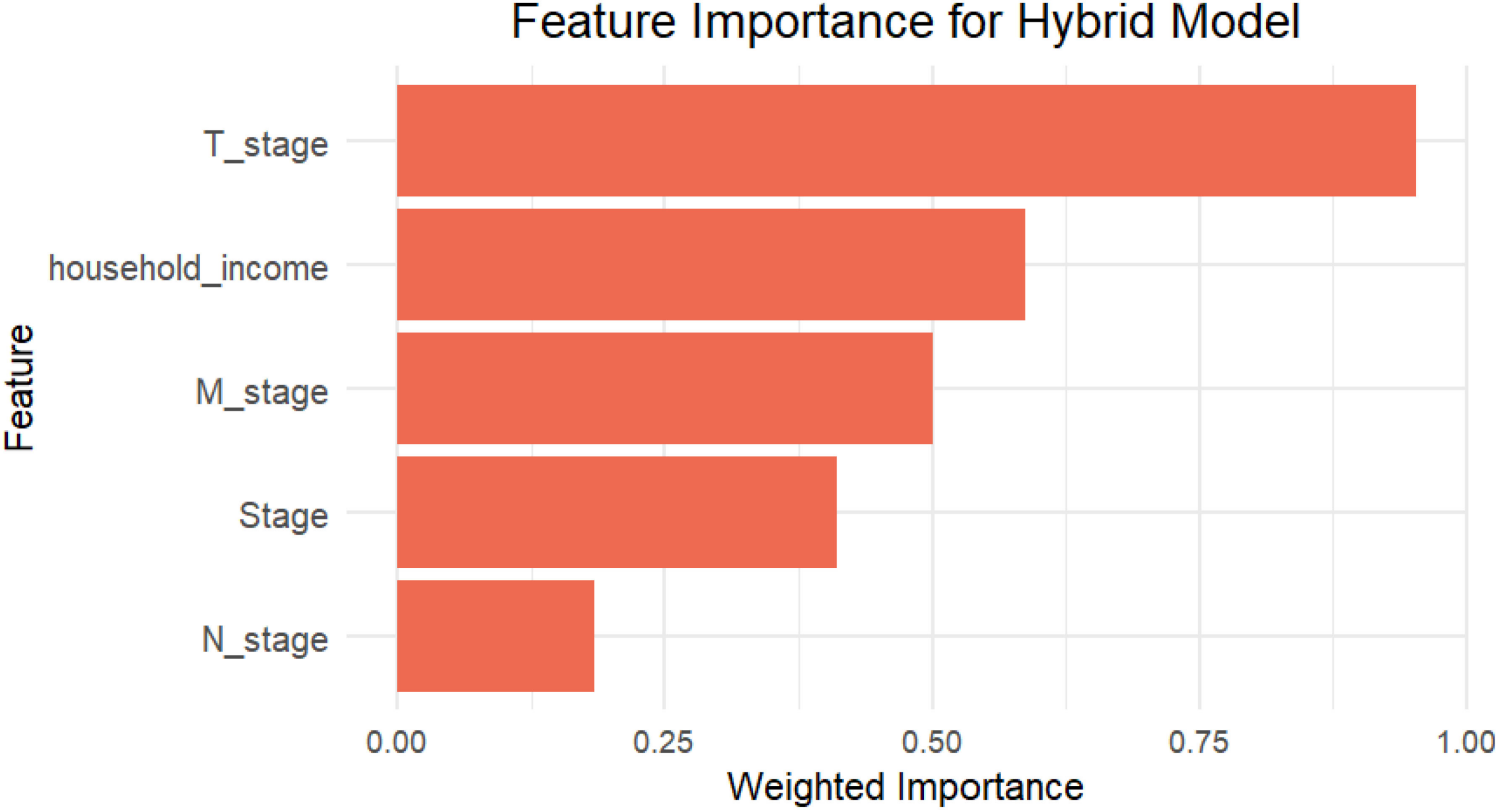
The bar plot shows the top five features contributing to the Hybrid model based on weighted importance scores, which collectively contributed most to the model’s survival risk estimation.

Using the importance scores from both models, the hybrid method was the most influential variable in both linear and non-linear situations (**Figure 5**). This blended feature significance analysis gives a comprehensive overview of the factors that influence survival outcomes, highlighting the hybrid model’s capacity to include different interactions from both models.

### 4.6 AUC-ROC analysis for the hybrid model

**Figure 6:**
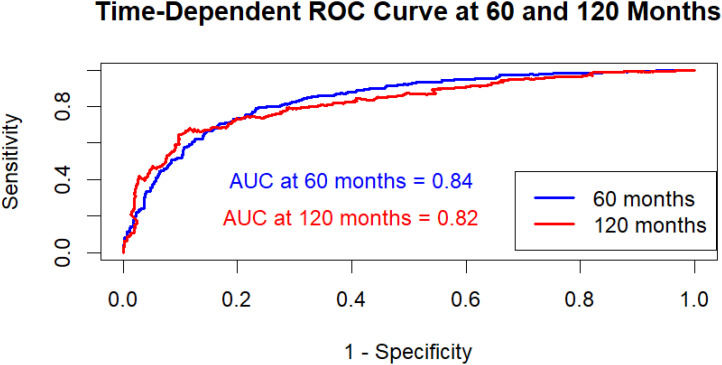
Time-dependent AUC-ROC curves at 60 and 120 months for the hybrid model. AUC at 60 months: 0.84 (95% CI: 0.81–0.87); AUC at 120 months: 0.82 (95% CI: 0.78–0.85). Confidence intervals computed using 1,000 bootstrap resamples.

An AUC-ROC curve was developed to further assess the hybrid model’s discriminative performance, showing the model’s capacity to differentiate between high-risk and low-risk patients over time (27). The time-dependent ROC curve shows how well the hybrid model predicts outcomes at two different time points: 60 and 120 months. The model has an AUC of 0.84 (95% CI: 0.81–0.87) at 60 months, indicating significant predictive power and improved capabilities in identifying individuals at increased risk of the occurrence. By 120 months, the AUC had dropped to 0.82 (95% CI: 0.78–0.85), showing a slight decline in accuracy but still high discriminatory capability. Overall, the model works well at both intervals, with just a small decline in precision as the time horizon increases. This shows that the model is reliable for both mid-term and long-term survival predictions, with shorter-term predictions being slightly more accurate. The ROC curve showed a distinct split between sensitivity and specificity, demonstrating that the model is useful in correctly identifying patients based on their survival outcomes (**Figure 6**). This AUC-ROC study validates the C-index results, showing the hybrid model’s higher classification performance over the separate Cox PH and RSF models.

## 5 Discussion

The present study discusses a hybrid machine-learning approach incorporating Cox proportional hazard with elastic net regularization, and random survival forest to predict cervical cancer survival. The objective was to use the capabilities of both models to enhance the accuracy and robustness of survival forecasts in clinical data, which frequently contains both linear and complicated non-linear interactions. The model outperformed either model on prediction accuracy, calibration, and classification, with a C-index of 0.82, an Integrated Brier Score (IBS) of 0.029, and an AUC-ROC of 0.84. These results highlight the hybrid model’s potential to improve clinical risk assessment and decision-making for cervical cancer patients.

The hybrid model’s improved performance suggests that survival outcomes are influenced by a combination of linear and nonlinear associations among variables. The Cox PH model, which is noted for its interpretability and ability to predict hazard ratios, was effective in discovering direct, linear correlations between variables and survival. These linear relationships are frequently related to well-known clinical risk variables, such as age, tumor stage, or biomarkers, which might have consistent and predictable impacts on survival rates. Elastic Net regularization, combining L1 (Lasso) and L2 (Ridge) penalties, improves feature selection and decreases multicollinearity concerns, hence minimizing overfitting and enhancing interpretability (14).

The RSF component increased the ability to simulate complicated, nonlinear relationships between variables, which are common in high-dimensional clinical data (18). Previous research has shown that RSF works well with survival data because it incorporates interactions between several factors without needing assumptions about their connections (28). By incorporating such interactions, RSF improves the model’s prediction accuracy and enhances the Cox PH model, which may overlook these non-linear effects. Our findings are consistent with previous research, which indicates that hybrid models that combine traditional survival models with machine-learning approaches can produce more accurate cancer prognostications (29).

Previous research in cancer survival modeling frequently depends on either Cox PH models or machine learning approaches such as RSF. Traditional Cox PH models are commonly employed in cancer studies due to their interpretability and ability to handle censored data. Zhang et al. (2013) proved the effectiveness of Cox PH models for discovering important predictors in survival analysis for different malignancies but with limited flexibility in dealing with non-linear interactions (30). The limitations of CoxPH models in high-dimensional data have sparked increased interest in RSF, which, according to Ishwaran et al. (2008), performs well in complicated datasets with interactions that cannot be predicted in advance (18).

In recent years, research has demonstrated that integrating linear models with machine-learning approaches can enhance forecast accuracy. Research in cancer and cardiovascular risk prediction, for example, has shown that hybrid techniques improve risk stratification and survival prediction compared to single-model approaches (31, 32). Our work expands on previous research by combining Cox PH and RSF, demonstrating that mixing linear and non-linear models may be extremely helpful. The weighted average strategy used in this work is also a simple but effective way to merge models with different properties, optimizing interpretability while improving prediction accuracy (33). In addition to this, Sundrani et al. (2021) used CoxPH models and decision-tree-based approaches to predict survival in breast cancer patients, improving predictive power by utilizing both linear and non-linear relationships (34). Furthermore, the hybrid model is consistent with previous efforts to merge classical survival models and machine learning. For example, Zhihua et al. (2018) presented a Cox-Bayesian hybrid to deal with missing data (35), whereas Yifei et al. (2013) used gradient boosting to increase the dependability of forecasting for a large-scale breast cancer dataset (36). Similarly, Yang et al. (2019) developed DeepCoxPH, which combined CoxPH with deep learning to improve risk categorization (37). Similarly, Pati et al. (2024) compared numerous hybrid machine learning algorithms for predicting breast cancer recurrence and found that these approaches outperformed single models in terms of accuracy and clinical value (38). Our hybrid strategy combines CoxPH and RSF, but employs Elastic Net regularization, which enables automatic feature selection and multicollinearity reduction on a SEER-based cervical cancer cohort, hence offering a unique balance of interpretability and predictive accuracy. Simsek et al. (2020) supports this approach by finding that hybrid models outperformed individual models in survival assessments (39).

The findings of this study have important implications for personalized medicine and clinical decision-making in cervical cancer. The hybrid model provides efficient risk classification by combining linear and nonlinear components, possibly helping clinicians to identify high-risk patients who may benefit from enhanced therapies or closer monitoring. For example, individuals designated as high-risk by the model may be prioritized for extra medications or follow-up tests. Meanwhile, low-risk individuals may avoid unneeded procedures, lowering both their physical stress and healthcare expenses. Also, the proposed hybrid model may be implemented into clinical processes to provide personalized survival risk estimations at the time of diagnosis or after therapy. The model, which effectively stratifies patients into high- and low-risk categories, can help clinicians modify follow-up intensity, select adjuvant medications, and prioritize patients for advanced procedures. Its interpretable component helps practitioners to understand major contributing factors, allowing for collaborative decision-making with patients. Furthermore, the CoxPH component’s interpretability increases the model’s clinical value by elucidating the correlations between various factors (such as tumor size, cancer stage, and age) and survival risk, allowing for evidence-based treatment decisions (40).

Furthermore, the hybrid model’s well-calibrated survival probabilities enable more accurate prognostic informs with patients giving them a better knowledge of predicted outcomes. This strategy is consistent with the aims of precision oncology, in which therapy is increasingly tailored to individual risk profiles (41). The AUC-ROC of 0.84 underlines the model’s classification accuracy, indicating that it might be used as a reliable tool for categorizing patients into high- and low-risk groups, which is important for treatment planning.

## 6 Limitations

While the hybrid model showed substantial improvements, some limitations should be considered when interpreting the findings of this study. First, the model was created and verified with data from the SEER database, which, while vast, is limited to the United States population. As a result, the model’s applicability to other groups, particularly those with different demographic and clinical features, has yet to be validated. Future research should aim to evaluate this model across a variety of diverse datasets to ensure its broad applicability (42).

Second, though the hybrid model improves prediction accuracy, it demands more computational capacity than Cox PH or RSF. This may restrict its usability in resource-constrained conditions or clinical contexts that lack high-performance computing machines. Optimizing the model to lower processing needs while maintaining accuracy might increase its viability for widespread clinical use (43).

An additional issue is the possible impact caused by unmeasured covariates. SEER data excludes some lifestyle factors (e.g., smoking, alcohol use, food), genetic information, and psychological variables, all of which might influence survival results. Including these factors in future studies of the model may improve its prediction accuracy and will provide an improved risk assessment (44).

## 7 Future study

This work provides opportunities for future research in several key areas. First, other machine learning techniques, such as deep neural networks, may be included to further increase the hybrid model’s accuracy in large and complex datasets. Neural networks may be able to identify complex patterns in the data, which might improve the hybrid model’s performance in high-dimensional survival analysis (45).

Second, prospective validation in actual clinical settings would offer important information on the effectiveness of the model in practice. Real-time survival predictions might be made possible by integrating the model with electronic health record (EHR) systems. This would enable doctors to dynamically modify risk assessments in response to new patient data. This strategy would allow for data-driven, flexible treatment planning adapted to each patient’s changing risk profile (46).

Furthermore, investigating model interpretability techniques such as Local Interpretable Model-agnostic Explanations (LIME) or Shapley additive explanations (SHAP) may improve the predictability of the hybrid model. These methods might boost clinician trust and enable more sophisticated decision-making by determining the role of specific factors in each prediction (47).

## 8 Conclusion

In conclusion, this work implies that a hybrid survival model integrating CoxPH Elastic Net Regularization and RSF improves predictive accuracy, robustness, and interpretability for cervical cancer patients. The hybrid model’s capacity to capture both linear and non-linear correlations makes it useful in clinical risk stratification, where precise survival forecasts are crucial for modified treatment planning. While more validation and improvement are required, this hybrid approach represents a potential step towards precision oncology, contributing to more effective, personalized cancer therapy. Future studies will focus on improving model generalizability and reducing computational complexity. Furthermore, using advanced interpretability approaches may improve the model’s transparency and clinical accessibility. In summary, this hybrid method provides a significant advancement towards precision oncology, with the potential for improving data-driven, patient-centered therapy in cervical cancer and beyond.

## Data Availability

The datasets presented in this study can be found in online repositories. The names of the repository/repositories and accession number(s) can be found below: The datasets analyzed during the current study are available in the SEER repository, https://seer.cancer.gov/.
